# Convex congruences

**DOI:** 10.1007/s00500-016-2306-8

**Published:** 2016-08-09

**Authors:** Ivan Chajda, Helmut Länger

**Affiliations:** 1 Faculty of Science, Department of Algebra and Geometry, Palacký University Olomouc, 17. listopadu 12, 771 46 Olomouc, Czech Republic; 2 Faculty of Mathematics and Geoinformation, Institute of Discrete Mathematics and Geometry, TU Wien, Wiedner Hauptstraße 8-10, 1040 Vienna, Austria

**Keywords:** Convex class, Convex congruence, Algebra with induced order, BCK-algebra, BCI-algebra

## Abstract

For an algebra ${\mathbf {A}}$ belonging to a quasivariety ${\mathcal {K}}$, the quotient ${\mathbf {A}}/\Theta$ need not belong to ${\mathcal {K}}$ for every $\Theta \in {{\mathrm{Con}}}~{\mathbf {A}}$. The natural question arises for which $\Theta \in {{\mathrm{Con}}}~{\mathbf {A}}, {\mathbf {A}}/\Theta \in {\mathcal {K}}$. We consider algebras ${\mathbf {A}}=(A,\rightarrow ,1)$ of type (2, 0) where a partial order relation is determined by the operations $\rightarrow$ and 1. Within these, we characterize congruences on ${\mathbf {A}}$ for which ${\mathbf {A}}/\Theta$ belongs to the same quasivariety as ${\mathbf {A}}$. In several particular cases, these congruences are determined by the property that every class is a convex subset of *A*.

It is well known that the class of BCK- resp. BCI-algebras forms a quasivariety which is not a variety, see Arai et al. (1966), Chajda and Kühr (2007), Imai and Iséki (1966), Iséki (1966) and Wroński (1983) for details. That BCK-algebras form a proper quasivariety was first shown in Wroński (1983). The reason is that although all but one axioms of BCK- resp. BCI-algebras are identities, the remaining one is only a quasiidentity. Hence, having such an algebra ${\mathbf {A}}=(A,\rightarrow ,1)$ from a quasivariety ${\mathcal {K}}$ and a congruence $\Theta$ on ${\mathbf {A}}$, the quotient algebra ${\mathbf {A}}/\Theta$ need not belong to ${\mathcal {K}}$ again. On every BCK- resp. BCI-algebra ${\mathbf {A}}=(A,\rightarrow ,1)$ one can define a partial order relation $\le$ by $x\le y$ if $x\rightarrow y=1$ ($x,y\in A$), see Imai and Iséki (1966) and Iséki (1966). It was proved in Traczyk and Zarȩbski (1985) that for a BCK-algebra ${\mathbf {A}}$ the algebra ${\mathbf {A}}/\Theta$ is a BCK-algebra again if and only if $\Theta$ is a so-called *convex congruence*, i.e., every class of $\Theta$ is a convex subset of the poset $(A,\le )$.

Our observation is that similar results hold for quasivarieties ${\mathcal {K}}$ properly including the class of BCK-algebras. It turns out that in general convexity of $\Theta$ need not be sufficient for ${\mathbf {A}}/\Theta \in {\mathcal {K}}$, but it is necessary in each case. We present a detailed inspection of conditions under which an algebra of the same similarity type as BCK- resp. BCI-algebras has a quotient belonging to the same quasivariety. These conditions can be formulated as identities and quasiidentities, but the corresponding quasivariety will not be explicitly mentioned.

Throughout the whole paper, we agree on the following conventions:

- The symbol ${\mathbf {A}}=(A,\rightarrow ,1)$ denotes a fixed algebra of type (2, 0).
- The symbol $\Theta$ denotes a fixed congruence on ${\mathbf {A}}$.
- The symbol $\le$ denotes the binary relation on *A* defined by
  1 $$\begin{aligned} x\le y\text { if and only if }x\rightarrow y=1. \end{aligned}$$
- The symbol $\le '$ denotes the binary relation on $A/\Theta$ defined by
  2 $$\begin{aligned}{}[x]\Theta \le '[y]\Theta \text { if and only if }[x]\Theta \rightarrow [y]\Theta =[1]\Theta .\quad \end{aligned}$$

Further, we will consider the following conditions:

3 $$\begin{aligned} \text {If }x,y\in & {} A\text { and }[x]\Theta \le '[y]\Theta ,\text { then there exists some }z \nonumber \\\in & {} [x]\Theta \text { with }z\le y. \end{aligned}$$

4 $$\begin{aligned} \text {If }x,y\in & {} A\text { and }[x]\Theta \le '[y]\Theta ,\text { then there exists some }z \nonumber \\\in & {} [y]\Theta \text { with }x\le z. \end{aligned}$$

In what follows we are interested in algebras ${\mathbf {A}}=(A,\rightarrow ,1)$ for which the relation defined by (1) is a partial order on *A*. Moreover, the following identities (5) – (8) will be considered in the paper:

5 $$\begin{aligned}&1\rightarrow x \approx x \end{aligned}$$

6 $$\begin{aligned}&x\rightarrow ((x\rightarrow y)\rightarrow y) \approx 1 \end{aligned}$$

7 $$\begin{aligned}&(x\rightarrow y)\rightarrow ((y\rightarrow z)\rightarrow (x\rightarrow z)) \approx 1 \end{aligned}$$

8 $$\begin{aligned}&(y\rightarrow z)\rightarrow ((x\rightarrow y)\rightarrow (x\rightarrow z)) \approx 1 \end{aligned}$$

First, we show some relations between the conditions just defined:

## Lemma 1

For the conditions (4)–(8) the following relationships hold:

(i) For any algebra ${\mathbf {A}}=(A,\rightarrow ,1)$ of type (2, 0) and any congruence $\Theta$ on it, (5) and (6) imply (4).
(ii) (4) does not imply (6).
(iii) (5) and (6) imply neither (7) nor (8).

## Proof

(i) If $a,b\in A$ and $[a]\Theta \le '[b]\Theta$ then
  $$\begin{aligned} (a\rightarrow b)\rightarrow b\in & {} [(a\rightarrow b)\rightarrow b]\Theta \\= & {} ([a]\Theta \rightarrow [b]\Theta ) \rightarrow [b]\Theta \\= & {} [1]\Theta \rightarrow [b]\Theta \\= & {} [1\rightarrow b]\Theta =[b]\Theta \end{aligned}$$
  and $$a\le (a\rightarrow b)\rightarrow b$$.
(ii) If ${\mathbf {A}}=(\{a,b,c,1\},\rightarrow ,1)$ with
  $$\begin{aligned} \begin{array}{c|cccc} \rightarrow &{}\quad a &{}\quad b &{}\quad c &{}\quad 1 \\ \hline a &{} \quad 1 &{} \quad c &{} \quad c &{} \quad 1 \\ b &{} \quad a &{} \quad 1 &{} \quad c &{} \quad 1 \\ c &{} \quad a &{} \quad b &{} \quad 1 &{} \quad 1 \\ 1 &{} \quad a &{} \quad b &{} \quad c &{} \quad 1 \end{array} \end{aligned}$$
  and $\Theta :=\{a\}^2\cup \{b,c,1\}^2$, then $\Theta \in {{\mathrm{Con}}}~{\mathbf {A}}$, $(A,\le )$ and $(A/\Theta ,\le ')$ are posets with the Hasse diagrams  and (4) holds, but (6) does not hold since
  $$\begin{aligned} a\rightarrow ((a\rightarrow b)\rightarrow b)= & {} a\rightarrow (c\rightarrow b) \\= & {} a\rightarrow b=c\ne 1. \end{aligned}$$
(iii) If ${\mathbf {A}}=(\{a,b,c,1\},\rightarrow ,1)$ with
  $$\begin{aligned} \begin{array}{c|cccc} \rightarrow &{}\quad a &{}\quad b &{}\quad c &{}\quad 1 \\ \hline a &{} \quad 1 &{}\quad b&{} \quad a &{}\quad 1 \\ b &{} \quad b &{}\quad 1 &{}\quad c &{}\quad 1 \\ c &{} \quad a &{}\quad b &{}\quad 1 &{}\quad 1 \\ 1 &{} \quad a &{}\quad b &{}\quad c &{}\quad 1 \end{array} \end{aligned}$$
  then ${\mathbf {A}}$ satisfies (5) and (6), but not (7) since
  $$\begin{aligned}&(a\rightarrow b)\rightarrow ((b\rightarrow c)\rightarrow (a\rightarrow c)) \\&\quad =b\rightarrow (c\rightarrow a)=b\rightarrow a=b\ne 1 \end{aligned}$$
  nor (8) since
  $$\begin{aligned}&(c\rightarrow a)\rightarrow ((b\rightarrow c)\rightarrow (b\rightarrow a)) \\&\quad =a\rightarrow (c\rightarrow b) =a\rightarrow b=b\ne 1. \end{aligned}$$
  $$\square $$

An interesting example of an algebra ${\mathbf {A}}=(A,\rightarrow ,1)$ for which the relation defined by (1) is a partial order is the (2, 0)-reduct of an integral commutative residuated poset. For the reader’s convenience, we repeat the definition of integral commutative residuated posets. An *integral commutative residuated poset* is an ordered quintuple ${\mathbf {P}}=(A,\le ,\cdot ,\rightarrow ,1)$ such that $(A,\le )$ is a poset, $(A,\cdot ,\rightarrow ,1)$ is an algebra of type (2, 2, 0) and the following holds for all $x,y,z\in A$:

- $(A,\cdot ,1)$ is a commutative groupoid with neutral element 1,
- $$x\le 1$$,
- $xy\le z$ if and only if $x\le y\rightarrow z$.

The following lemma is well known:

## Lemma 2

If ${\mathbf {P}}=(A,\le ,\cdot ,\rightarrow ,1)$ is an integral commutative residuated poset and $a,b\in A$ then the following hold:

(i) $a\le b$ if and only if $a\rightarrow b=1$.
(ii) $a\le b$ if and only if $a\le 1\rightarrow b$.
(iii) ${\mathbf {P}}$ satisfies (5) and (6).

## Proof

(i) The following are equivalent:
  $$\begin{aligned} a=1 a\le b,\;1\le a\rightarrow b,\;a\rightarrow b=1. \end{aligned}$$
(ii) The following are equivalent:
  $$\begin{aligned} a\le b,\;a1\le b,\;a\le 1\rightarrow b. \end{aligned}$$
(iii) ${\mathbf {P}}$ satisfies (5) since $1\rightarrow b\le b$ (take $a=1\rightarrow b$ in (ii)) and $b\le 1\rightarrow b$ (take $a=b$ in (ii)) and it satisfies (6) since every one of the following assertions implies the next one:
  $$\begin{aligned} x\rightarrow y\le & {} x\rightarrow y,\;(x\rightarrow y)x\le y,\;x(x\rightarrow y) \\\le & {} y,\;x\le (x\rightarrow y)\rightarrow y,\;(6). \end{aligned}$$
  $$\square $$

## Remark 3

Lemma 2 justifies the notation $\le$ in integral commutative residuated posets since $\le$ is connected with $\rightarrow$ exactly as in (1).

Recall that ${\mathbf {A}}$ is called a BCK*-algebra* if the relation defined by (1) is a partial order on *A* with greatest element 1 and (5) – (7) are satisfied. Since $x\le 1$ we have $x\rightarrow 1\approx 1$ according to (1) and, moreover,

$$\begin{aligned} x\rightarrow (y\rightarrow x)\approx & {} 1\rightarrow (x\rightarrow (y\rightarrow x)) \\\approx & {} (y\rightarrow 1)\rightarrow ((1\rightarrow x)\rightarrow (y\rightarrow x))\approx 1. \end{aligned}$$

according to (5) and (7). Usually, ${\mathbf {A}}$ is called a BCK-algebra if it satisfies (6), (7) and (9) – (11) (cf. Chajda et al. (2007)):

9 $$\begin{aligned} x\rightarrow x\approx & {} 1 \end{aligned}$$

10 $$\begin{aligned} x\rightarrow 1\approx & {} 1 \end{aligned}$$

11 $$\begin{aligned} x\rightarrow y= & {} y\rightarrow x=1 \Rightarrow x=y \end{aligned}$$

Now the equivalence of both axiom systems follows easily by using the results in Chajda et al. (2007). The algebra ${\mathbf {A}}$ is called a BCI*-algebra* if the relation defined by (1) is a partial order on *A* and (5), (6) and (8) are satisfied.

The following example shows that there exist integral commutative residuated posets $(P,\le ,\cdot ,\rightarrow ,1)$ such that $(P,\rightarrow ,1)$ is neither a BCK- nor a BCI-algebra.

## Example 4

If $A=\{a,b,c,1\}$, $(A,\le )$ denotes the chain $a<b<c<1$ and binary operations $\cdot$ and $\rightarrow$ on *A* are defined by

$$\begin{aligned} \begin{array}{c|cccc} \cdot &{}\quad a &{}\quad b &{}\quad c &{}\quad 1 \\ \hline a &{} \quad a&{} \quad a &{} \quad a &{} \quad a \\ b &{} \quad a&{} \quad a &{} \quad b &{} \quad b \\ c &{} \quad a&{} \quad b &{} \quad b &{} \quad c \\ 1 &{} \quad a&{} \quad b &{} \quad c &{} \quad 1 \end{array} \quad \text {and}\quad \begin{array}{c|cccc} \rightarrow &{}\quad a &{}\quad b &{}\quad c &{}\quad 1 \\ \hline a &{} \quad 1 &{} \quad 1 &{} \quad 1 \quad &{} \quad 1 \\ b &{} \quad b &{} \quad 1 &{} \quad 1 \quad &{} \quad 1 \\ c &{} \quad a &{} \quad c &{} \quad 1 \quad &{} \quad 1 \\ 1 &{} \quad a &{} \quad b &{} \quad c \quad &{} \quad 1 \end{array} \end{aligned}$$

then $(A,\le ,\cdot ,\rightarrow ,1)$ is an integral commutative residuated poset which is neither a BCK-algebra since

$$\begin{aligned} (c\rightarrow b)\rightarrow ((b\rightarrow a)\rightarrow (c\rightarrow a))= & {} c\rightarrow (b\rightarrow a) \\= & {} c\rightarrow b=c\ne 1 \end{aligned}$$

contradicting (7) nor a BCI-algebra since

$$\begin{aligned} (b\rightarrow a)\rightarrow ((c\rightarrow b)\rightarrow (c\rightarrow a))= & {} b\rightarrow (c\rightarrow a) \\= & {} b\rightarrow a=b\ne 1 \end{aligned}$$

contradicting (8). Moreover, $(A,\cdot )$ is not a monoid since

$$\begin{aligned} (bc)c=bc=b\ne a=bb=b(cc). \end{aligned}$$

## Lemma 5

If ${\mathbf {P}}=(A,\le ,\cdot ,\rightarrow ,1)$ is an integral commutative residuated poset and $\Theta \in {{\mathrm{Con}}}(A,\cdot ,\rightarrow ,1)$ then (3) holds.

## Proof

If $a,b\in A$ and $[a]\Theta \le '[b]\Theta$ then

$$\begin{aligned} (a\rightarrow b)a\in [(a\rightarrow b)a]\Theta= & {} ([a]\Theta \rightarrow [b]\Theta )[a]\Theta \\= & {} [1]\Theta [a]\Theta =[1a]\Theta =[a]\Theta \end{aligned}$$

and $$(a\rightarrow b)a\le b$$ since $$a\rightarrow b\le a\rightarrow b$$. $$\square $$

## Definition 6

The congruence $\Theta$ is called *convex* if $a,b,c\in A$, $a\le b\le c$ and $a\,\Theta \,c$ imply $a\,\Theta \,b$.

## Lemma 7

The relations defined by (1) and (2) satisfy the following implications:

(i) If $a,b\in A$ and $a\le b$ then $[a]\Theta \le '[b]\Theta$.
(ii) If $\le$ is reflexive so is $\le '$.
(iii) If $\le '$ is antisymmetric, then $\Theta$ is convex.

## Proof

(i) If $a,b\in A$ and $a\le b$ then $[a]\Theta \rightarrow [b]\Theta =[a\rightarrow b]\Theta =[1]\Theta$.
(ii) This follows from (i).
(iii) If $a,b,c\in A$, $a\le b\le c$ and $a\,\Theta \,c$ then according to (i) we have $[a]\Theta \le '[b]\Theta \le '[c]\Theta =[a]\Theta$ which implies $[a]\Theta =[b]\Theta$, i.e., $a\,\Theta \,b$.$\square$

## Definition 8

The algebra ${\mathbf {A}}$ is called an *algebra with induced order* if the relation defined by (1) is a partial order on *A*. The algebra ${\mathbf {A}}/\Theta$ is called an *algebra with induced order* if the relation defined by (2) is a partial order on $A/\Theta$.

## Theorem 9

If ${\mathbf {A}}/\Theta$ is an algebra with induced order, then $\Theta$ is convex.

## Proof

This follows from (iii) of Lemma 7. $\square$

Theorem 9 shows that convexity of $\Theta$ is necessary for ${\mathbf {A}}/\Theta$ to be an algebra with induced order.

## Example 10

If $${\mathbf {A}}=(\{a,b,1\},\rightarrow ,1)$$ with

$$\begin{aligned} \begin{array}{c|ccc} \rightarrow &{}\quad a &{}\quad b &{}\quad 1 \\ \hline a &{}\quad 1 &{} \quad 1 &{} \quad 1 \\ b &{}\quad a &{} \quad 1 &{} \quad 1 \\ 1 &{}\quad a &{} \quad a &{} \quad 1 \end{array} \end{aligned}$$

and $\Theta :=\{b\}^2\cup \{a,1\}^2$ then $\Theta \in {{\mathrm{Con}}}~{\mathbf {A}}$, $(A,\le )$ is a poset with $a\le b\le 1$, $\Theta$ is not convex, and $(A/\Theta ,\le ')$ is not a poset since $\{b\}\le '\{a,1\}\le '\{b\}$.

Next we present two conditions under which the property of ${\mathbf {A}}/\Theta$ to be an algebra with induced order is equivalent to the convexity of $\Theta$.

## Theorem 11

If ${\mathbf {A}}$ is an algebra with induced order and (4) holds, then ${\mathbf {A}}/\Theta$ is an algebra with induced order if and only if $\Theta$ is convex.

## Proof

Let $a,b,c\in A$. First we show that if (4) holds and $\Theta$ is convex, then $\le '$ is antisymmetric. Hence assume $\Theta$ to be convex and (4) to hold. Further, assume $[a]\Theta \le '[b]\Theta \le '[a]\Theta$. According to (4), there exists an element *d* of $[b]\Theta$ with $a\le d$. Since $[d]\Theta =[b]\Theta \le '[a]\Theta$, there exists an $e\in [a]\Theta$ with $d\le e$ according to (4). Now $a\le d\le e$ and $a\,\Theta \,e$. Since $\Theta$ is convex, we have $a\,\Theta \,d$ and hence $[a]\Theta =[d]\Theta =[b]\Theta$ showing antisymmetry of $\le '$. Next we show that under assumption (4), transitivity of $\le$ implies transitivity of $\le '$. Hence assume $\le$ to be transitive and (4) to hold. Further, assume $[a]\Theta \le '[b]\Theta \le '[c]\Theta$. According to (4), there exists an $f\in [b]\Theta$ with $a\le f$. Since $[f]\Theta =[b]\Theta \le '[c]\Theta$, there exists an element *g* of $[c]\Theta$ with $f\le g$ according to (4). Now $a\le f\le g$ and hence $a\le g$ which implies $[a]\Theta \le '[g]\Theta =[c]\Theta$ according to Lemma 7 showing transitivity of $\le '$. The rest follows from Lemma 7. $\square$

That assumption (4) in Theorem 11 cannot be dropped is shown by the following

## Example 12

If $${\mathbf {A}}=(\{a,b,1\},\rightarrow )$$ with

$$\begin{aligned} \begin{array}{c|ccc} \rightarrow &{}\quad a &{}\quad b &{}\quad 1 \\ \hline a &{} \quad 1 &{} \quad 1 &{} \quad 1 \\ b &{} \quad b &{} \quad 1 &{} \quad 1 \\ 1 &{} \quad b &{} \quad b &{} \quad 1 \end{array} \end{aligned}$$

and $\Theta :=\{a\}^2\cup \{b,1\}^2$ then $\Theta \in {{\mathrm{Con}}}~{\mathbf {A}}$, $(A,\le )$ is a poset with $a\le b\le 1$, $\Theta$ is convex, $(A/\Theta ,\le ')$ is not a poset since $\{a\}\le '\{b,1\}\le '\{a\}$ and (4) does not hold since $[1]\Theta \le '[a]\Theta$, but $1\not \le a$.

In an analogous way as it was already done for Theorem 11, one can prove

## Theorem 13

If ${\mathbf {A}}$ is an algebra with induced order and (3) holds then ${\mathbf {A}}/\Theta$ is an algebra with induced order if and only if $\Theta$ is convex.

## Theorem 14

If ${\mathbf {P}}=(A,\le ,\cdot ,\rightarrow ,1)$ is an integral commutative residuated poset and $\Theta \in {{\mathrm{Con}}}(A,\cdot ,\rightarrow ,1)$, then ${\mathbf {P}}/\Theta :=(A/\Theta ,\le ',\cdot ,\rightarrow ,[1]\Theta )$ is an integral commutative residuated poset if and only if $\Theta$ is convex.

## Proof

According to Lemma 5, (3) holds and according to Lemmata 1 and 2, (4) holds. Now assume $\Theta$ to be convex. According to Remark 3 and Theorem 11, $(A/\Theta ,\le ')$ is a poset. Moreover, $(A/\Theta ,\cdot ,\rightarrow ,[1]\Theta )$ is an algebra of type (2, 2, 0) and $(A/\Theta ,\cdot ,[1]\Theta )$ is a commutative groupoid with neutral element $[1]\Theta$. Let $a,b,c\in A$. Then $[a]\Theta \le '[1]\Theta$ according to Lemma 7. If $[a]\Theta [b]\Theta \le [c]\Theta$, then $[ab]\Theta \le [c]\Theta$ and hence according to (4) there exists an element *d* of $[c]\Theta$ with $ab\le d$ whence $a\le b\rightarrow d$ which finally implies

$$\begin{aligned}{}[a]\Theta \le '[b\rightarrow d]\Theta =[b]\Theta \rightarrow [d]\Theta =[b]\Theta \rightarrow [c]\Theta \end{aligned}$$

according to Lemma 7. If, conversely, $[a]\Theta \le '[b]\Theta \rightarrow [c]\Theta$, then $[a]\Theta \le '[b\rightarrow c]\Theta$ and hence according to (3) there exists an $e\in [a]\Theta$ with $e\le b\rightarrow c$ whence $eb\le c$ which finally implies

$$\begin{aligned}{}[a]\Theta [b]\Theta =[e]\Theta [b]\Theta =[eb]\Theta \le '[c]\Theta \end{aligned}$$

according to Lemma 7. The rest follows from Remark 3 and Theorem 11. $\square$

## Lemma 15

The following implications hold:

(i) (5) and (7) imply that the relation defined by (1) is reflexive and transitive.
(ii) (5) and (8) imply that the relation defined by (1) is reflexive and transitive.

## Proof

Let $$a,b,c\in A$$.

(i) We have
  $$\begin{aligned} a\rightarrow a= & {} 1\rightarrow (a\rightarrow a) \\= & {} (1\rightarrow 1)\rightarrow ((1\rightarrow a)\rightarrow (1\rightarrow a))=1, \end{aligned}$$
  i.e., $a\le a$, and if $a\le b\le c$ then
  $$\begin{aligned} a\rightarrow c= & {} 1\rightarrow (a\rightarrow c)=1\rightarrow (1\rightarrow (a\rightarrow c))= \\= & {} (a\rightarrow b)\rightarrow ((b\rightarrow c)\rightarrow (a\rightarrow c))=1, \end{aligned}$$
  i.e., $$a\le c$$.
(ii) We have
  $$\begin{aligned} a=1\rightarrow a\le (1\rightarrow 1)\rightarrow (1\rightarrow a)=1\rightarrow a=a \end{aligned}$$
  and if $a\le b\le c$ then
  $$\begin{aligned} a\rightarrow c= & {} 1\rightarrow (a\rightarrow c)=1\rightarrow (1\rightarrow (a\rightarrow c))= \\= & {} (b\rightarrow c)\rightarrow ((a\rightarrow b)\rightarrow (a\rightarrow c))=1, \end{aligned}$$
  i.e., $$a\le c$$.$$\square $$

## Theorem 16

The following implications hold:

(i) If the relation defined by (1) is antisymmetric and ${\mathbf {A}}$ satisfies (5) and either (7) or (8), then ${\mathbf {A}}$ is an algebra with induced order.
(ii) If ${\mathbf {A}}$ satisfies (5) and (6) and either (7) or (8), then ${\mathbf {A}}/\Theta$ is an algebra with induced order if and only if $\Theta$ is convex.

## Proof

(i) This follows from Lemma 15.
(ii) This follows from Lemmata 1 and 7 and from the proof of Theorem 11.$\square$

## Corollary 17

The following implications hold:

(i) (cf. Traczyk and Zarȩbski 1985) If ${\mathbf {A}}$ is a BCK-algebra then ${\mathbf {A}}/\Theta$ is a BCK-algebra if and only if $\Theta$ is convex.
(ii) If ${\mathbf {A}}$ is a BCI-algebra then ${\mathbf {A}}/\Theta$ is a BCI-algebra if and only if $\Theta$ is convex.

## Proof

These follow from Lemmata 1 and 7 and Theorem 11. $\square$
